# Report of the Premier African Organisation for Research and Training in Cancer Virtual Genomics Conference held from 18–20 February 2021

**DOI:** 10.3332/ecancer.2021.1253

**Published:** 2021-06-21

**Authors:** Hannah NG Ayettey Anie, Solomon O Rotimi, Pedro Fernandez, Belmira Rodrigues, Omolara Aminat Fatiregun, Nwamaka Lasebikan, Skye Wilson, Khatija Osman, Lavender Nyoni, Lisa Newman, Folakemi T Odedina

**Affiliations:** 1 National Radiotherapy Oncology and Nuclear Medicine Centre, Guggisberg Avenue, PO Box KB 319, Korlebu Teaching Hospital, Accra, Ghana, West Africa; 2 Department of Biochemistry, Covenant University, Ota, Nigeria; 3 Division of Urology, Stellenbosch University, Cape Town, South Africa; 4 African Organisation for Research and Training in Cancer (AORTIC), 1st Floor, Birkdale 1, River Park, Gloucester Road, Mowbray, Cape Town, South Africa; 5 Lagos State University College of Medicine/Teaching Hospital, Lagos, Nigeria; 6 University of Nigeria Teaching Hospital Enugu, Enugu, Nigeria; 7 NewYork-Presbyterian/Weill Cornell Medical Center and Weill Cornell Medicine, New York, USA; 8 University of Florida, Research and Academic Center at Lake Nona, FL, USA

**Keywords:** Africa, African Organization for Research and Training in Cancer (AORTIC), cancer, genomics, genetics

## Abstract

The rapidly rising cancer burden and mortality rate in Africa are in contrast to the increase in cancer survivorship in Europe and North America. Genomic medicine has contributed to the rise in survival and has facilitated precision cancer control. However, there is a shortage of African representation in genomic databases, even for cancers that disproportionately affect Africans. To improve this outlook and address research in genomics and genetics relevant to Africa and people of African descent, the African Organisation for Research and Training in Cancer (AORTIC), under the Research Committee’s auspices, organised the Cancer Genomic Conference. The conference aimed to develop a roadmap for cancer genomics research to control the continent’s increasing cancer burden. Presentations at the conference revealed that: (1) Africa is made up of a highly heterogeneous group of people with diverse ethnic groups, (2) Very few African countries have been the focus of cancer genomics research, (3) Cancer exacts a heavy burden on global populations across the African diaspora with obvious genetic variants and cancer disparities and (4) There are differences in the contribution of genetics by race or ancestry and these differences are likely due to evolutionary genetics, contextual factors and genomic architecture. The importance of data security, ethics and integrity of the African genomics data was emphasised. The implementation of the conference highlights will provide the bedrock for pharmacogenomics to guide treatment decisions for cancer in Africa. The conference concluded with the formation of an AORTIC Special Interest Group on cancer genomics. It is the goal of this group to drive the implementation of this Conference’s outcomes.

## Introduction

Africa faces a unique and rapidly rising cancer burden, with incidence and mortality projected to double by 2040 [1]. This rapidly growing mortality rate in Africa contrasts with the increase in cancer survivorship in Europe and North America [2]. A major factor contributing to this increase in survival is the advent of genomics medicine which has facilitated precision cancer control in those populations [2]. Cancer is primarily a genomic disease. Therefore, it is not surprising that the understanding of genetic or genomics has been utilised to improve clinical outcomes in different populations outside of Africa.

The recognition of cancer as a genomic disease has led to concerted extensive projects like the American Association for Cancer Research Project Genomics Evidence Neoplasia Information Exchange and The Cancer Genome Atlas. These projects are designed to help decipher clinically actionable genomic alterations in different cancers. Although these projects impact is far-reaching, their utilisation for cancer control in Africa is limited by the shortage of African representation in these databases, even for cancers that disproportionately affect Africans. At best, Africans have only contributed 3% of genetics studies [3]. Attempts to address these gaps in genomics training and research have resulted in initiatives like Human Heredity and Health in Africa (H3Africa), which, however, does not have cancer as a priority disease [4]. To address this and other gaps limiting cancer genomics research and implementation in Africa and create increased awareness and a platform for nuanced discussions on genomics-driven cancer control in Africa, the African Organisation for Research and Training in Cancer (AORTIC) Research Committee initiated the African Cancer Genomic Conference.

## Conference summary

This premier genomics conference was held virtually from the 18 to 20 of February 2021, under the AORTIC Research committee’s auspices, which has the unique role of promoting and spearheading research aligned activities within the organisation and Africa as a whole. This conference was also part of the committee’s effort at fostering dissemination of African research through scientific conferences, peer-reviewed journals, traditional media and social media.

**Figure 1.**
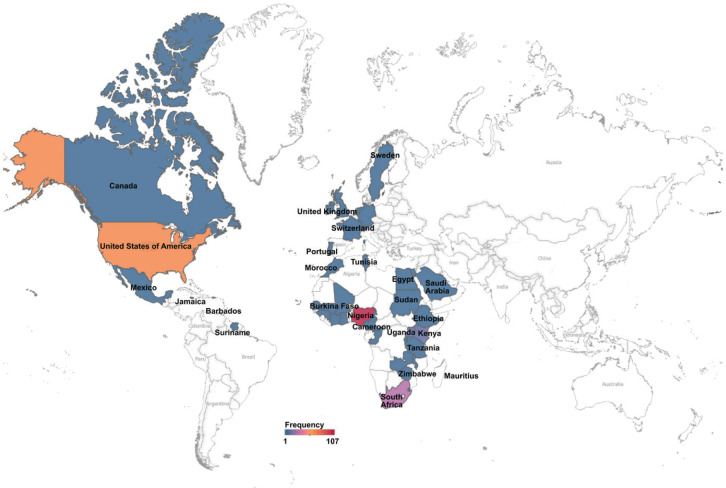
Heat map showing the proportion of registered participants for the Conference. Most Africa-affiliated participants were from Nigeria and South Africa, while the US had the highest number of non-Africa affiliated participants.

The Cancer Genomics Virtual Conference was held under the theme ‘African Genomic Diversity, A Roadmap to Global Equity in Cancer Control’. This was a 2-day conference which was immediately followed on the third day by an invitation-only meeting for further deliberation on the theme. The conference’s vision was to address basic science and clinical research in cancer genomics and genetics relevant to Africa and people of African descent. The conference had a mission of bringing genomics experts in Africa and the diaspora to encourage discussion on genomics and how it relates to Africa in current research and management of cancer. An important outcome was to develop a roadmap for cancer genomics research that will address the continent’s increasing cancer burden. This was to help us achieve our goal of developing a network of scientists to lead genomic science in Africa through an AORTIC Cancer Genomics Special Interest Group (SIG). The conference attracted 303 registered participants from 42 countries globally (Figure 1). Twenty-five of the countries were from Africa, with the realization of 80 new AORTIC members.

As this growing field of research is critically important to cancer training, research and clinical management in Africa, we brought different genomics experts from the United States of America, Africa and the diaspora to facilitate discussion. The conference also highlighted what the status quo was for research in Africa. Keynote speakers included, Professor John Carpten, Professor and Chair of the Department of Translational Genomics, Keck School of Medicine, University of Southern California; Professor Lisa Newman, the Chief of Breast Surgery and Director of the Interdisciplinary Breast Programme at the Weill Cornell Medicine/New York-Presbyterian hospital network; Professor Timothy Rebbeck, the Vincent L. Gregory Professor of Cancer Prevention at the Harvard TH Chan School of Public Health and Dana Farber Cancer Institute and Professor Olufunmilayo Olopade, Walter L. Palmer Distinguished Service Professor and Associate Dean for Global Health at The University of Chicago Medical Center.

The conference commenced on day 1 with a welcome address by the conference planning committee Chair, Dr Hannah Ayettey Anie, who mentioned the importance and perfect timing of the genomics conference being held in Africa. The AORTIC President, Dr Abubakar Bello, welcomed participants, highlighting the conference’s importance with respect to basic science, cancer genomics and genetics research. He specifically mentioned the unique relevance of cancer genomics to Africa and people of African descent, noting that few researchers have taken an interest in African genomic diversity. He concluded by saying that this conference will stimulate research in Africa by Africans in genomics. The AORTIC Research Committee Chair, Professor Folakemi Odedina then elaborated on the strategic plan of the AORTIC research committee, which has a charge to provide scientific leadership and direction for cancer research in Africa through AORTIC activities. She highlighted the committee’s focus on the research challenges in Africa and its objectives to meet and mitigate these challenges, which include: Creation of larger collaborative groups, establishing regional biobanks, development of pilot grants to assist junior faculty, developing a framework for expanding research infrastructure and funding, bridging the knowledge gap in clinical research methodology in Africa, equipping African institutions to be clinical trial-ready and gaining the trust of the population for medical research. The genomics conference was a specific goal of the research dissemination group of the committee, which worked in collaboration with the SIG to organise the conference. Professor Odedina, in her concluding comments, said that ‘All roads lead to Africa for genetics’, noting that studying populations of African descent was not only about Africa but the whole world, underscoring the importance of human variation, the evolutionary history of variants and the heritability and genetic architecture of complex diseases like cancer resulting in the development of a new clinical tool for diverse populations.

The opening lecture on cancer genomics in Africa was delivered by Dr Solomon Rotimi, AORTIC Research Committee Co-chair and member of the conference planning committee. He gave a very interesting disposition on African ancestry and the definition of an African. In his submission, studying the human evolution and history of the world lent credence to the clear fact that we are all Africans, referring to the whole world population having emanated from ancestors on the African continent at some point in world history. He mentioned that Africa comprises a highly heterogeneous group of people with diverse ethnic groups and separated by colonial borders. Greater than 90% of human genetic diversity had not yet been studied, with only 2.4% of genomic studies representing Africans at present. He specifically noted that very few countries like Northern Africa countries, South Africa, Nigeria and Ghana had been the focus of research in Africa, stressing collaboration.

The first keynote speaker for the day was Professor John Carpten, who delivered a lecture on the topic ‘An overview of genomic cancer studies in populations of Sub-Saharan African ancestry’. His lecture focused on work done on cancer genome science in Black populations, reiterating that cancer exacts a heavy burden on global populations across the African diaspora with obvious genetic variants and cancer disparities. He further underscored the significant influence of social factors such as financial toxicities and access on these disparities. Professor Carpten stressed that there was limited investigation into tumour biology differences across racial and ethnic groups, hence an absence of data. These studies will broaden our understanding of cancer’s complexity to best approach disease management more effectively for all our patients. He discussed current developments in racial disparity studies in different types of cancers, including breast, prostate, colorectal, ovarian, endometrial cancer, leukaemia and multiple myeloma, suggesting that these studies will uncover health disparity-related factors and provide more depth in understanding the disease aetiology.

Dr Pedro Fernandez, an AORTIC Research Committee member and member of the genomics planning committee, immediately followed this lecture with a talk on ethical considerations in genomics. He spoke on the importance of this in genomics, focusing on the ethical guidelines and benchmarks for multinational clinical research, including research ethics principles, a fair selection of study populations, favourable risk-benefit, the importance of independent review, informed consent, respect for recruited participants and study communities and lessons learnt from collaborative research. He stated that health research as a discipline had been beset with scandals and atrocities, mentioning Henrietta Lacks’ story and origin of the HeLa cell line. Health research ethics had, therefore, established itself as a distinct field to address such abuses. A significant point he highlighted was that data could be collected, stored and analysed in Africa! He further emphasised the importance of bioinformatics and data science moving forward.

Dr Melissa Davis of the Weill Cornell Medical College then delivered a lecture on genomic data management and security, highlighting concerns in genomic research for data security, ethics and integrity. She outlined data security issues related to participants, including privacy, confidentiality, ethical protection and investigator-facing considerations related to data storage, access and integrity of the analysis. Dr Davis noted that several studies had demonstrated the vulnerability of human genomic data if they are insufficiently protected hence the importance of legal protection and data storage. She outlined the components of a bioinformatics workflow and the importance of gatekeepers of access to data.

Professor Kosj Yamoah of the Moffitt Cancer Center and Research Institute deliberated on genomic data acquisition and proper utilisation in the context of oncologic care. In his submission, he defined precision oncology as a medical approach that proposes preventing and treating disease based upon a person’s unique genetic makeup and lifestyle habits. He defined genomic medicine noting that cancers of a given histologic diagnosis are genomically heterogeneous and caused by somatic mutations, not genetic polymorphisms. He shed light on the importance of prognostic and predictive biomarkers and their use in managing our patients using prostate cancer as an example.

Day 1 conference proceedings ended with a series of pre-recorded oral abstract presentations. These focused on basic science, translational and clinical research in genomics, bringing out the critical importance of cancer research in African populations and studying genetic diversity, as well as the power of international partnership and sharing of technology and resources. A poster session on Twitter followed this.

Day 2 commenced with the second keynote lecture of the conference delivered by Professor Lisa Newman, who centred her lecture on ‘Oncologic anthropology: High-risk triple-negative breast cancer and African ancestry’. This lecture focused on the global distribution of locally adaptive traits and African Americans’ breast cancer burden, presenting with higher mortality, advanced stage and socioeconomic disparities. The Duffy null allele was explicitly mentioned, highlighting studies done on its prominence in African American populations and its relation to malaria development. Of special importance was the realisation that the Duffy null allele is a likely determinant of the triple-negative breast cancer status in women of African descent. Professor Newman also added that there were possibly other related determinants in a multifactorial process.

Professor Timothy Rebbeck delivered the third keynote lecture for the conference titled ‘Genetics of prostate cancer in Sub-Saharan Africa’. His lecture forecasted Africa as a hot spot for prostate cancer and leading in prostate cancer mortality compared to Caucasian men. He noted that disparities are the result of complex, multifactorial inputs. Highlights on the Men of African Descent and Carcinoma of the Prostate (MADCaP) network, which has sought to study the disease’s epidemiology and understand the low and high penetrance genes associated with prostate cancer in this population of men, was discussed. In summary, Professor Rebbeck said, ‘prostate cancer in Africa is heavily genetically influenced and less epidemiologically influenced’. There are differences in the contribution of genetics by race or ancestry, and these differences are likely due to evolutionary genetics, contextual factors and genomic architecture. He concluded that large, well-annotated cohorts with tissue are required to extend these findings for clinical translation.

Professor Olufunmilayo Olopade, in her lecture on ‘Cancer genomics in oncology: The example of the Breast Cancer (BRCA) genes,’ shared her journey as a medical oncologist and clinical geneticist with emphasis on work done on BRCA genes in Nigerian women. Her dream was what African oncology would look like in 2030. She advocated for studies on the oral and written history of western civilisation, studying the distribution of heritable breast cancer, social barriers to diagnosis and treatment of breast cancer and the interface of science with culture. ‘There is the need to motivate people to change behaviour and additionally improve access to genetic testing in Africa’ were her comments.

Dr Peter Kingham from the Memorial Sloan Kettering Cancer Center focused on the clinical application of genomics research and colorectal cancer. He drew attention to the growing problem of cancer in low- and middle-income countries. Comparing patients in Nigeria to Caucasian patients in the United States of America, it was realised that there were fewer patients with Adenomatous Polyposis Coli (APC) mutations and hence more of them had aggressive pathology. Furthermore, a higher percentage of Rat Sarcoma (RAS) gene mutations and low frequency of Wingless-related Integration Site (WNT) pathway alterations suggest that this pathway was not the dominant driver of Microsatellite Stable tumorigenesis in this population. He reiterated the importance of including genetic studies into comprehensive cancer control programmes implying that with the unique mutational profile of colorectal cancer in Nigeria, caution should be taken before generalising therapeutic trial results produced in high-income countries.

On improving cancer care through pharmacogenomics, Dr Julie Johnson, Dean of the College of Pharmacy, University of Florida, discussed the clinical potential of pharmacogenetics and the concept of precision medicine or personalised medicine. She emphasised the trial-and-error approach in pharmacogenetics, harping on the fact that pharmacogenetics often focused on germline variation. It has also been observed from studies that pharmacogenetic variations exist among people of African ancestry and Caucasians. Genotype-guided approaches to drug therapy management could improve clinical outcomes, especially in cancer patients who have great potential for benefit from the use of pharmacogenomics to guide treatment decisions.

The second day’s final thought-provoking lecture was delivered by Dr Howard McLeod, Medical Director, Geriatric Oncology Consortium and Professor at the University of South Florida. His lecture centred on diagnostic applications – Introduction to next-generation sequencing and precision oncology. Variation in response to therapy, unpredictable toxicity and the optimisation of tumour control and patient support were the highlights of his lecture. It became clear that to adequately manage a cancer patient, germline and somatic mutations both have to be focused on. Dr McLeod accentuated that next-generation sequencing accorded objective guidance from molecular changes in the tumour, cautioning that access to drugs was of the essence if we were to do genomics effectively. He implored AORTIC to play a forefront role in this feat, stressing that the benefit is in the therapy, not in the sequencing: The sequence is a means to an end and not the end in itself. Further, he asked the question, ‘Who has the clinicians back after ordering personalised medicine services when it comes to interpretation and drawing valid conclusions of results?’ The development of resistance mutations and the setback it brings in the ongoing treatment was not left out of the discussion. He concluded by admonishing us not to shrink the tumour but help the patient, likewise not just do science, but science that helps people!

The day ended with the second batch of pre-recorded oral abstract presentations on the first day, focusing on genomic studies being done in Africa. Dr Abubakar Bello, AORTIC President, gave the closing remarks and brought the conference to a close.

The third day, 20 of February 2021, was by invitation only and focused on drawing a roadmap for cancer genomics research with an overarching objective of developing the African cancer genome atlas. This meeting took the form of four breakout sessions held for about an hour, each with different sets of questions to be addressed. Breakout group one focused on driving intercountry research and training partnership among African cancer investigators to strengthen research funding and improve political will to support cancer genomics research on the continent. The suggestions that emerged to overcome the limitations were to: (1) Organise more local-regional conferences to facilitate collaboration among researchers in Africa interested in genomics; (2) Institutionalise and strengthen the AORTIC Cancer Genomics SIG as a fulcrum for seeking funding and international collaboration; (3) Work on capacity building to improve Africa’s research environment, political will and commercial research from Africa and (4) Seek to protect African samples and make research on the continent more Afrocentric. Breakout group one also worked on the goals and objectives for the cancer genomics SIG, which are:

1. Driving inter-country research and training partnership among African cancer investigators. This is to strengthen research funding and improve political will to support cancer genomics research on the continent.
2. Development of a network system within Africa and with non-African senior cancer genomics researchers for training and research exchanges.
3. Improving training curricula to include or emphasise cancer genomics in medical and biomedical training programmes in African institutions.
4. Encourage digitisation of biobanking and development of virtual repositories to publicise the bio-specimen available across the continent. The integration of the virtual repositories will be facilitated and coordinated by AORTIC.
5. Establishment of Genomic educational sessions during the AORTIC biennial meeting. The genomics working group’s leadership will actively seek training funding like R13 and Civilian Research and Development Foundation (CRDF) to support the training.
6. Promotion of cancer genomics research in Africa through an annual special issue in a reputable journal or AORTIC journal.
7. Promotion of genomic diagnosis and genetic counselling at Oncology centres across Africa.

Breakout group two focused on improving training curricula, emphasising cancer genomics in medical and biomedical training programmes in African institutions and encouraging digitisation of biobanking and development of repositories to publicise the available biospecimen across the continent. Following the discussion, the following goals were set: (1) Train and include cancer genomics in both undergraduate and postgraduate levels with more emphasis at the postgraduate level; (2) Encourage collaboration between medical scientists and clinicians on cancer genomics; (3) Engage more in trans lational pharmacogenetics and pharmacogenomics research; (4) Encourage rapid career progression for young researchers in their area of interest; (5) Encourage multidisciplinary collaborations vertically and horizontally to ensure availability and accessibility of essential cancer medicines; (6) Leverage available grants and existing programmes for the development of cancer genomics in Africa and (7) Publish research findings on cancer genomics in African journals and other platforms.

The recommendations for the encouragement of digitisation of biobanking and development of repositories to publicise the biospecimen included the following: (1) Invest in training and developing a growing number of African biostatisticians, bioinformaticians and data scientists to analyse increasing amounts of stored/banked data; (2) Incorporate bio-banking into grant applications and train principal investigators with the expertise to apply for such grants; (3) Buy into private sector developed data centres in Africa; (4) Encourage public-private partnerships in biobanking while exercising caution against biospecimen commercialisation and profit-making by private partners; (5) Take advantage of philanthropic donations and strive to sustain existing facilities in Africa and (6) Digitise biobanks and promote virtual storage to overcome the massive barrier of inconsistent power supply. Partnership with the American Society of Clinical Pathologists and technological companies with non-commercial interest will help digitise biobanks, virtual storage and electronic data transfer.

Breakout group three discussed issues concerning the establishment of a genomics educational session during AORTIC biennial meetings and, consequently, actively seek funding for training by applying for extramural conference and research training grants. The next discussion point was on promoting cancer genomics research in Africa through an annual special issue in a reputable journal or proposed AORTIC journal. Recommendations drawn from these discussions were: (1) Establish a genomics educational session. Funding will be sought for this pre-conference training by applying for the United States (US) National Institutes of Health Conference (R13) grant with collaborators in the US; (2) Promote cancer genomics research in Africa through an annual special issue. This issue will focus on research papers and operational matters, including the development of registries, strengthening existing population-based registries and biorepositories and (3) Train African journalists to effectively cover cancer genomics research and clinical practice through brief courses for journalists.

Breakout group four discussion was primarily centred on promoting genomic diagnosis and genetic counselling at oncology centres across Africa. After a vibrant discussion on the topic, the recommendations were: (1) Improve oncology professionals’ training and sensitisation on genomics, in collaboration with the AORTIC Education and Training Committee; (2) Incorporate the teaching of genomic diagnosis and genetic counselling into our institutions’ training curricula at the undergraduate and postgraduate levels; (3) Improve the accessibility of genetic testing for the populace and introduce this cautiously, considering the socio-cultural barriers in African populations. There was a proposal to do feasibility studies, situational analysis and meta-analysis on already existent studies to assess the populace’s knowledge base on genomics and (4) Train genetic counsellors, oncologists, psycho oncologists and nurses in genetic counselling and finally ensure that genetic counsellors form part of the multidisciplinary team in institutions.

Recommendations from all four breakout sessions were presented to the whole group. This was immediately followed by a launch of the AORTIC Cancer Genomics SIG by Ms Belmira Rodrigues, who provided an overview on the requirements for SIGs in AORTIC. AORTIC President closed the meeting after the accomplishments of the conference objectives.

## Conflicts of interest

The authors have declared no competing interests.
